# Incidence and Timing of Epstein–Barr Virus Whole Blood DNAemia in Epstein–Barr Virus‐Mismatched Adult and Pediatric Solid Organ Transplant Recipients

**DOI:** 10.1111/tid.70042

**Published:** 2025-04-29

**Authors:** Catherine Burton, Curtis Mabilangan, Jutta Preiksaitis

**Affiliations:** ^1^ Division of Infectious Diseases, Department of Pediatrics University of Alberta Edmonton Alberta Canada; ^2^ Division of Infectious Diseases Department of Medicine, University of Alberta Edmonton Alberta Canada

**Keywords:** Epstein–Barr virus, post‐transplant lymphoproliferative disorders, transplant

## Abstract

**Background:**

Epstein–Barr virus (EBV) viral load (VL) monitoring is recommended post‐transplant for EBV‐mismatched (donor EBV seropositive/recipient EBV seronegative) solid organ transplant (SOT) recipients as a component of post‐transplant lymphoproliferative disorder (PTLD) prevention, but the optimal frequency and timing of EBV VL monitoring remains unknown.

**Methods:**

In this retrospective cohort study, we investigated the incidence and timing of whole blood EBV DNAemia in EBV‐mismatched adult and pediatric SOT recipients, who had EBV VL monitoring as part of a pre‐emptive approach to PTLD prevention to optimize monitoring algorithms. We explored associations between donor‐acquired EBV DNAemia (DA‐EBV), defined as EBV DNAemia within 1 year post‐transplant, and donor and recipient characteristics, and determined the proportion who developed PTLD.

**Results:**

We analyzed 257 D^+^/R^−^ recipients (kidney *n* = 64, heart *n* = 75, liver *n* = 93, lung *n* = 25); 126/257 (49.0%) developed DA‐EBV at a median of 83 days (Q1–Q3: 50–130 days) post‐transplant. Incidence of DA‐EBV varied by organ and was highest in liver (62.4%) and lowest in heart recipients (28.0%). PTLD was diagnosed in 38/257 (14.8%) EBV‐mismatched recipients, 25/162 (15.4%) children, and 13/95 (13.7%) adults. DA‐EBV was uncommon in recipients less than 6 months old (3/29, 10.3%) and among recipients less than 12 months with donors less than 12 months (2/29, 6.9%); possible mechanisms of protection other than recipient passive maternal antibody and false‐positive donor serostatus are discussed.

**Conclusion:**

Monitoring for DA‐EBV should be focused on months 2–6 post‐transplant. Less frequent whole blood EBV VL monitoring is likely a safe option in recipients less than 6 months old and recipients 6–12 months old with donors less than 12 months old.

## Graphical abstract

“In this observational study of 257 EBV‐mismatched (EBV D^+^/R^−^) adult and pediatric solid organ transplant (SOT) recipients, we investigate the incidence and timing of whole blood EBV DNAemia as well as associations between donor‐acquired EBV DNAemia and donor and recipient age, and organ transplanted to provide evidence to optimize post‐transplant EBV viral load monitoring in post‐transplant lymphoproliferative disorder‐prevention protocols.”

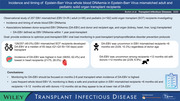

**Abbreviations**
CA‐EBV: community‐acquired EBV
D^+^/R^−^
: donor positive/recipient negative
DA‐EBV: donor‐acquired EBV
DLBCL: diffuse large B‐cell lymphoma
EBER: EBV‐encoded small RNAs
EBV: Epstein–Barr virus
HR: hazard ratio
LOD: limit of detection
PTLD: post‐transplant lymphoproliferative disorder
Q1–Q3: quartile 1 to quartile 3
SOT: solid organ transplant
VL: viral load
WB: whole blood

## 1 Introduction

Post‐transplant lymphoproliferative disorders (PTLD) are responsible for significant morbidity and mortality in solid organ transplant (SOT) recipients, with 5‐year cumulative incidence rates of 0.6%–16%, varying by allograft type and age at transplant [1, 2, 3]. Most PTLD in the first few years post‐transplant is associated with Epstein–Barr virus (EBV) infection. EBV‐naive recipients, infected with EBV transmitted from donor organs, are at particularly high risk [1, 4–7].

International guidelines recommend EBV viral load (VL) monitoring in donor positive/recipient negative (D^+^/R^−^) recipients, combined with interventions that may lower VL as a pre‐emptive strategy for PTLD prevention, but the optimal frequency and timing of EBV VL monitoring remains unknown [1, 8–11]. Detailed descriptions of allograft‐specific cumulative incidence of EBV DNAemia, donor and recipient characteristics influencing EBV transmission from donor organs, and determining the ongoing risk of community‐acquired EBV infection (CA‐EBV) for those escaping donor‐transmitted infections are critical to inform and tailor the optimal frequency and duration of EBV VL monitoring in PTLD prevention protocols.

At our institution, a pre‐emptive PTLD prevention approach targeting EBV‐mismatched SOT recipients was implemented in 2002. This included whole blood (WB) EBV VL monitoring for the first year post‐transplant, with intervention such as reduction in immunosuppression at the transplant physician's discretion when VL rose significantly. During the study period, antiviral prophylaxis was also recommended for EBV‐mismatched recipients for 14 weeks post‐transplant.

Our objective was to investigate the incidence and timing of EBV DNAemia in EBV mismatched (D^+^/R^−^) adult and pediatric SOT recipients, investigate associations between EBV DNAemia and donor and recipient age, and organ transplanted, and determine the proportion of EBV‐mismatched recipients who developed PTLD to provide evidence to optimize EBV monitoring protocols.

## 2 Materials and Methods

### 2.1 Study Population

We identified all adult and pediatric (<17 years) recipients who were EBV seronegative (R^−^) and received an EBV seropositive (D^+^) organ (kidney, pancreas, liver, heart, lung, and multivisceral) at the University of Alberta Hospital/Stollery Children's Hospital (Edmonton) between January 1, 2002 and July 31, 2016. Patients who died within 1 month of transplant or had no EBV VL monitoring were excluded. Patient data, including age, sex, organ type, transplant date, and pre‐transplant D/R EBV serostatus, were obtained from a prospectively maintained transplant database. Organs were grouped as follows: kidney–pancreas with kidneys, multivisceral with liver, and heart–lung with lung. Patients with more than one transplant event were tracked from the first mismatched transplant. This study was approved by the University of Alberta Health Research Ethics Board (HREB Pro00082134).

### 2.2 EBV Serology and VL Testing

All recipients and local donors were tested before transplant for EBVCA IgG and EBNA‐1 IgG using an enzyme immunoassay (Captia Trinity Biotech, Bray, Ireland). Distant donor serology was provided by the Human Organ Procurement and Exchange program (assays unknown). Donors and recipients were considered EBV seropositive if EBVCA IgG or EBNA‐1 IgG was positive. Seropositive infants, less than 12 months old, were considered EBV seronegative if recipients and seropositive if donors (highest risk scenario).

In 2002, we implemented post‐transplant EBV VL monitoring in WB weekly from Week 4 to Week 20, and then monthly from Month 6 to Month 12. In 2008, a baseline EBV VL on Day 1 post‐transplant was added. Additional “for cause” EBV VL testing was ordered at the attending physician's discretion. We estimated each patient's protocol compliance as the number of samples tested in the first year post‐transplant, divided by the expected number of samples, adjusted for early transfer out to other centers. From January 2002 to February 2016, EBV DNA was quantified in WB using an in‐house developed real‐time PCR assay [limit of detection (LOD) 500 copies/mL = ∼250 IU/mL by cross‐referencing] [12]. After March 2016, a commercial assay was used (RealStar PCR, Altona Diagnostics, Hamburg, Germany [LOD 550 IU/mL]) [13]. EBV DNAemia was defined as the detection of EBV DNA above the assay LOD. Because VL rises rapidly in primary EBV infection, peaks well above LOD, and persists beyond adjacent monitoring intervals used in our study, the relative sensitivity of the two assays we used is unlikely to have significantly impacted the timing and detection of EBV DNAemia. EBV DNAemia occurring in the first year post‐transplant was considered donor‐acquired EBV (DA‐EBV); DNAemia occurring after 1 year post‐transplant was considered community‐acquired (CA‐EBV). EBV VL data were collected from each patient's transplant date until July 31, 2017.

### 2.3 Antiviral Prophylaxis

During the study period, all adult and almost all pediatric EBV‐mismatched recipients were expected to receive antiviral prophylaxis, with ganciclovir/valganciclovir, for 14 weeks post‐transplant. From 2010 onwards, antiviral prophylaxis was not routinely recommended in EBV‐mismatched pediatric heart recipients, but was still recommended in all other pediatric and all adult recipients. Recipients may have received antiviral prophylaxis/a longer duration of antiviral prophylaxis for cytomegalovirus (CMV) prevention.

### 2.4 Immunosuppression

Details of induction and maintenance immunosuppressive regimens by organ are presented in Table S1.

### 2.5 PTLD Data

PTLD data and all VL data were collected until December 31, 2018. Diagnosis date, pathology, EBV‐encoded small RNAs (EBER) status, and treatment for first episode PTLD cases were extracted from a prospectively maintained PTLD database. Pathology diagnoses were classified according to the WHO 2016 classification system [14]. One case of an EBV‐associated smooth muscle tumor was included.

### 2.6 Statistical Analysis

In this retrospective cohort study, we analyzed associations between the incidence of EBV DNAemia and recipient and donor age group and organ group using cumulative incidence curves. Cumulative incidence curves were compared using log‐rank test. Pairwise comparisons were adjusted using Benjamini–Hochberg procedure. Hazard ratios (HR) were obtained using purposeful Cox proportional hazards model building. For the multivariable models, variables that were statistically significant on univariable analysis with a *p*‐value of less than 0.2 were included in addition to variables that were considered a priori to be clinically significant. Patients were censored at the study end date, last follow‐up date, transfer out date, or deceased date and at 2 years post‐transplant if no EBV DNAemia detection event occurred. Variables were assessed for interaction and confounding. All analyses were performed using R 3.6.3.

## 3 Results

### 3.1 Patient Population

We identified 279 unique recipient EBV‐mismatched transplant events. After excluding 22 recipients, nine who died within 1 month of transplant and 13 without EBV VL testing, 257 were analyzed: 64 kidney (3/64 = kidney/pancreas), 93 liver (6/93 = multivisceral), 75 heart and 25 lung (3/25 = heart/lung) recipients. Recipient demographics by organ group are described in Table S2. The median EBV VL monitoring compliance was 91.6% (quartile 1 to quartile 3 [Q1–Q3] = 58.3%–100%) and was highest in kidney and liver recipients and lowest in lung recipients (Table S2). Antiviral prophylaxis would have been recommended for 241/257 recipients (93.8%); only 16 pediatric heart transplant recipients after 2010 would not have had anti‐viral prophylaxis recommended for either CMV or EBV.

### 3.2 Incidence and Timing of EBV DNAemia

Overall, 126/257 (49.0%) recipients developed EBV DNAemia within 1 year of transplant, and median time to DA‐EBV was 83 days (Q1–Q3 = 50–130 days) (Table 1). Time to first positive EBV VL is summarized in Figure S1. Given the long incubation period of 32–49 days for EBV in immunocompetent subjects, we carefully examined detectable EBV VLs occurring early (<3 weeks) post‐transplant. A 10‐month‐old liver recipient, with negative EBVCA and EBNA IgG 32 days pre‐transplant, had EBV DNAemia on the day of transplant, suggesting recent infection. A 0.8‐month‐old heart recipient with a 2.5‐month‐old donor had a single low‐level, detectable EBV VL 2 weeks post‐transplant, but 1 week later and all subsequent EBV VL monitoring to 1 year post‐transplant was negative; the single positive EBV VL was thought to be a false‐positive result.

**TABLE 1:** Incidence of whole blood EBV DNAemia in first 2 years post‐transplant by age and organ.

	*N* (%) with EBV DNAemia <1 year post‐Tx (DA‐EBV)	Median (Q1–Q3 =) days to first EBV detection among DA‐EBV	*N* (%) with EBV DNAemia >1 year post‐Tx (CA‐EBV)	*N* (%) without EBV DNAemia by 2 years post‐Tx	Total recipients
**EBV DNAemia by recipient age**					
<6 months	3 (10.3%)	74 (44–79)	2 (6.9%)	24 (82.8%)	29 (100%)
6–12 months	28 (45.9%)	72 (44–89)	6 (9.8%)	27 (44.3%)	61 (100%)
12 months to 5 years	24 (68.6%)	75 (54–97)	2 (5.7%)	9 (25.7%)	35 (100%)
5–17 years	23 (62.2%)	72 (36–137)	3 (8.1%)	11 (29.7%)	37 (100%)
≥17 years	48 (50.5%)	102 (73–148)	6 (6.3%)	41 (43.2%)	95 (100%)
**EBV DNAemia by organ**					
Kidney	39 (60.9%)	87 (50–109)	5 (7.8%)	20 (31.2%)	64 (100%)
Liver	58 (62.4%)	73 (49–93)	4 (4.3%)	31 (33.3%)	93 (100%)
Heart	21 (28.0%)	97 (58–159)	7 (9.3%)	47 (62.7%)	75 (100%)
Lung	8 (32.0%)	160 (147–249)	3 (12.0%)	14 (56.0%)	25 (100%)
**Total recipients**	126 (49.0%)	83 (50–130)	19 (7.4%)	112 (43.6%)	257 (100%)

### 3.3 EBV DNAemia by Donor and Recipient Age, Sex, and Organ

**FIGURE 1:**
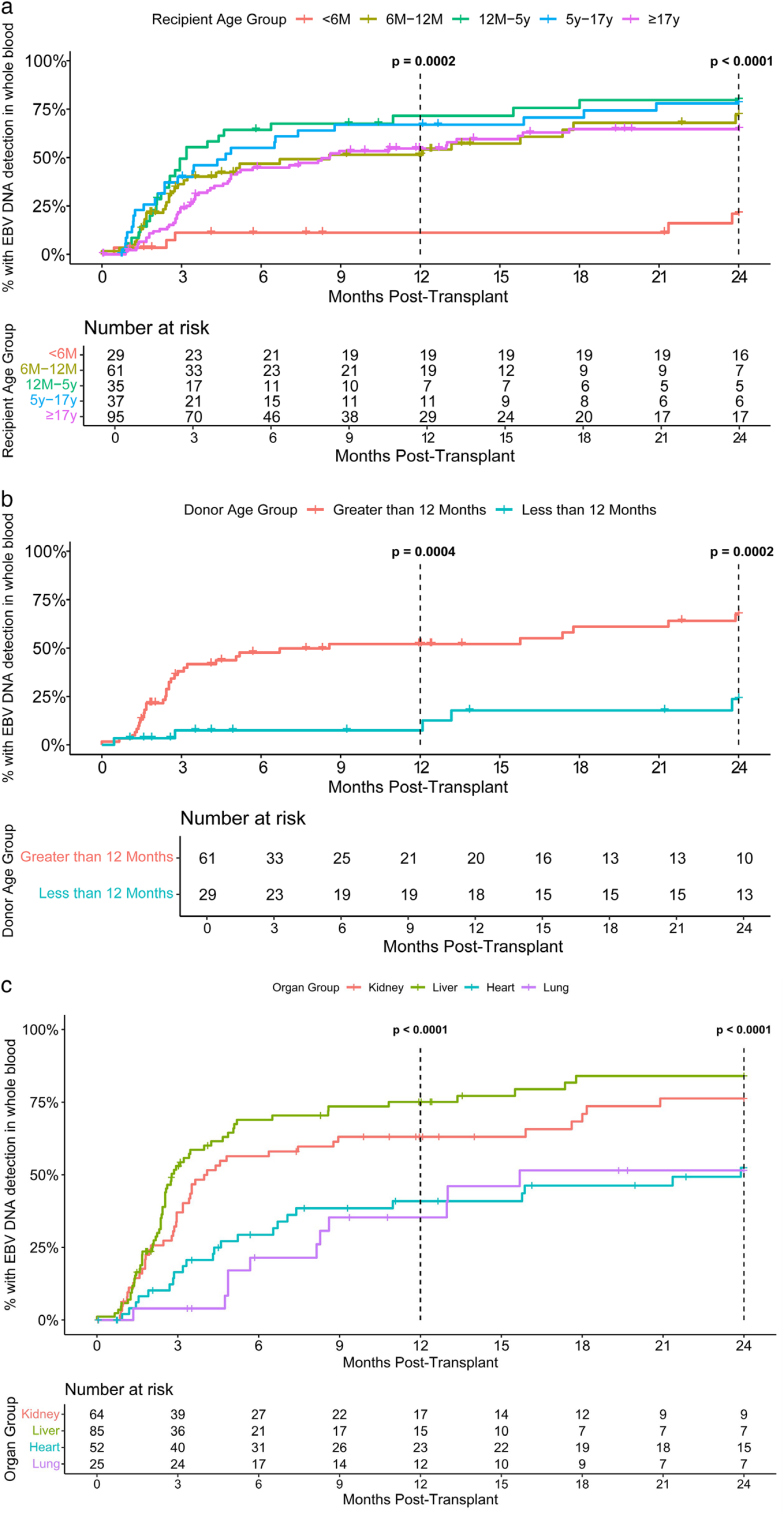
(a) Cumulative incidence of whole blood EBV DNAemia by recipient age at transplant. (b) Cumulative incidence of whole blood EBV DNAemia in recipients <12 months old, by donor age group. (c) Cumulative incidence of whole blood EBV DNAemia by organ group, excluding donors age <12 months old.

DA‐EBV by recipient age, sex, and organ transplanted is summarized in Table 1. Incidence of DA‐EBV was significantly lower in EBV‐mismatched recipients less than 6 months old compared with all other recipient age groups (Figure 1a, *p* = 0.0002). Only three of 29 recipients less than 6 months old had EBV DNAemia detected within 1 year post‐transplant. A 4.6‐month‐old heart recipient with a 2.9‐month‐old donor had EBV DNAemia at 12 weeks post‐transplant, and a 3.6‐month‐old liver recipient with a 3.8‐year‐old donor had EBV DNAemia at 10 weeks post‐transplant. Both recipients were EBV seropositive pre‐transplant, but were considered seronegative due to age. The third case was the false‐positive described above. Of the 29 recipients less than 6 months old, 18 had donors who were less than 12 months old, 16 of whom were less than 6 months old, and two were 6–12 months old. Of the 11 recipients less than 6 months old with donors ≥12 months old, who are more likely to be truly EBV‐infected, only one of eight (12.5%) with positive pre‐transplant EBV serology, presumably due to maternal antibody, developed DA‐EBV. Interestingly, none of the three with negative pre‐transplant EBV serology developed DA‐EBV. In contrast, among 50 recipients 6–12 months old with donors ≥12 months old, 14 of 26 (53.8%) who had positive pre‐transplant EBV serology and 14 of 24 (58.3%) who had negative pre‐transplant serology developed DA‐EBV.

The incidence of DA‐EBV in infants less than 12 months old with donors less than 12 months old was significantly lower compared to infants with donors older than 12 months old (Figure 1b, *p* = 0.0004). Only two of 29 recipients less than 12 months old with donors less than 12 months old had DA‐EBV (described above); one was likely false‐positive.

Figure 1c illustrates the incidence of DA‐EBV by organ, excluding the 31 recipients with donors less than 12 months old, as infant donor serology is unreliable. The distribution of infant donors was highly skewed to heart (*n* = 23) and liver (*n* = 8) recipients. The incidence of DA‐EBV was significantly higher among liver and kidney recipients compared with heart and lung recipients (*p* < 0.05 for adjusted pairwise comparisons for kidney vs. heart [*p* = 0.009], kidney vs. lung [*p* = 0.012], liver vs. heart [*p* < 0.001], and liver vs. lung [*p* < 0.001]).

We performed multivariable Cox regression analysis investigating the effects of recipient and donor age, recipient sex, and organ transplanted (using kidney as a reference group) on DA‐EBV (Table 2). We excluded donors and recipients less than 12 months old due to unreliable serology. Being a liver recipient was significantly associated with increased risk of DA‐EBV, while being a lung recipient was associated with decreased risk. Increasing recipient age was associated with decreased risk, and recipient male sex was associated with an increased risk of DA‐EBV. As compliance with the EBV VL monitoring varied significantly by organ, we repeated this multivariable regression including only recipients with ≥50% compliance with EBV VL monitoring. Only the increased risk of DA‐EBV in liver recipients and the decreased risk with increasing recipient age remained (data not shown).

**TABLE 2:** Multivariable Cox regression: Donor‐acquired EBV DNAemia in donors and recipients over 12 months of age.

Variable	Univariable HR (95% CI)	Multivariable HR (95% CI)
**Age**		
Recipient age	**0.985 (0.974–0.996)** ^**^	**0.986 (0.973–0.998)** ^*^
Donor age	1.012 (0.999–1.025)	1.011 (0.996–1.026)
**Sex**		
Recipient female	1 (Ref)	1 (Ref)
Recipient male	1.391 (0.914–2.116)	**1.583 (1.022–2.452)** ^*^
Donor female	1 (Ref)	1 (Ref)
Donor male	0.823 (0.548–1.236)	0.866 (0.564–1.331)
**Organ** ^a^		
Kidney	1 (Ref)	1 (Ref)
Liver	**1.803 (1.129–2.878)** ^*^	**1.648 (1.007–2.697)** ^*^
Heart	0.607 (0.334–1.101)	0.669 (0.351–1.277)
Lung	**0.354 (0.165–0.758)** ^**^	**0.422 (0.195–0.914)** ^*^

Abbreviations: CI, confidence interval; HR, hazard ratio; Ref, reference group.

^a^ Organs are grouped as follows: kidney–pancreas with kidney, multivisceral with liver, and heart‐lung with lung.

^**^ *p* < 0.01; ^*^ *p* < 0.05.

### 3.4 EBV Transmission Patterns From a Single EBV‐Seropositive Donor to Multiple EBV‐Seronegative Recipients

Ten EBV‐seropositive donors donated organs to multiple EBV‐seronegative recipients. Four of seven donors more than 12 months old donated organs to two seronegative recipients each; all eight recipients developed DA‐EBV. There was no EBV transmission from an adolescent donor to a heart and kidney recipient. In the other two cases, transmission was not consistent. An adult donor transmitted to a liver but not to a heart recipient. An adolescent donor transmitted to a kidney and liver recipient, but not to a heart recipient.

Three donors less than 12 months old donated organs to two seronegative recipients each; none of the six recipients had DA‐EBV.

### 3.5 PTLD

Of 257 EBV‐mismatched recipients, 38 (14.8%) developed PTLD (Table 3); 25 (25/162, 15.4%) cases occurred in pediatric recipients, nine of 40 (22.5%) in those at “low risk” for DA‐EBV (recipients <6 months or 6–12 months with donors <12 months) versus 16/122 (13.1%) in other pediatric recipients and 13/95 (13.7%) in adult recipients. The timing, pathology and EBER status of the PTLD cases, EBV VL histories, and time from first EBV detection to PTLD are summarized in Figure S2a,b. Twelve PTLD cases (31.6%) were diagnosed within a year post‐transplant; 30 cases (78.9%) by Year 4. The median time post‐transplant to PTLD was 37.0 months (Q1–Q3 = 33.5–43.2) for “low risk” DA‐EBV infants, 14.0 months (Q1–Q3 = 8.9–29.7) in other pediatric recipients, and 15.7 months (Q1–Q3 = 11.5–72.4) in adults. EBV VL was detected prior to or at the time of PTLD diagnosis in 24/29 recipients with EBER‐positive PTLD, with a median (Q1–Q3) time from transplant to first EBV detection of 144 (69–479) days. Nine recipients never had documented detectable EBV VL prior to or within 4 weeks after the diagnosis of PTLD; five had EBER‐positive PTLD, and four had unknown EBER status (Figure S2a). Of the five EBER‐positive cases without detectable EBV VL, one had Hodgkin lymphoma (kidney recipient) and three had diffuse large B‐cell lymphoma (DLBCL) (two lung and one heart recipient); one of the lung transplant recipients with DLBCL had PTLD isolated to the pleura. A kidney recipient had polymorphic PTLD isolated to the central nervous system, with EBV VL detectable only in cerebrospinal fluid. All five had a negative WB EBV VL within 15 days before or after PTLD diagnosis; testing was done prior to rituximab therapy in all cases.

**TABLE 3:** Characteristics of EBV‐mismatched recipients who developed PTLD.

	Adult (*N* = 13)	Pediatric (*N* = 25)	Total (*N* = 38)
**Organ**			
Heart	0 (0.0%)	13 (52.0%)	13 (34.2%)
Lung	9 (69.2%)	0 (0.0%)	9 (23.7%)
Liver	1 (7.7%)	9 (36.0%)	10 (26.3%)
Kidney	3 (23.1%)	3 (12.0%)	6 (15.8%)
**Sex**			
Male	4 (30.8%)	12 (52.0%)	17 (44.7%)
Female	9 (69.2%)	13 (48.0%)	21 (55.3%)
**Age at transplant (years)** ^a^	38.4 (30.8–45.4)	0.81 (0.48–2.5)	2.65 (0.65–29.2)
**Age at PTLD diagnosis (years)** ^a^	43.2 (31.1–51.5)	3.9 (2.9–5.1)	5.9 (3.4–30.7)
**Time from transplant to PTLD (months)** ^a^	15.7 (11.5–72.4)	25.8 (10.7–42.2)	24.5 (10.9–45.2)
**Early PTLD (<1 year post‐transplant)**	4 (30.8%)	8 (32.0%)	12 (31.6%)
**Pathology**			
Nondestructive PTLD	0 (0.0%)	7 (28.0%)	7 (18.4%)
Polymorphic	1 (7.7%)	4 (16.0%)	5 (13.2%)
DLBCL	9 (69.2%)	8 (32.0%)	17 (44.7%)
Burkitt lymphoma	0 (0.0%)	2 (8.0%)	2 (5.3%)
Peripheral T‐cell lymphoma, NOS	0 (0.0%)	1 (4.0%)	1 (2.6%)
Classical Hodgkin lymphoma	2 (15.4%)	0 (0.0%)	2 (5.3%)
Smooth muscle tumor^b^	0 (0.0%)	1 (4.0%)	1 (2.6%)
PTLD NOS	1 (7.7%)	2 (8.0%)	3 (7.9%)
**Tumor EBER status**			
Positive	9 (69.2%)	20 (80.0%)	29 (76.3%)
Negative	3 (23.1%)	0 (0.0%)	3 (7.9%)
Unknown	1 (7.7%)	5 (20.0%)	6 (15.8%)
**Total PTLD**	13 (100%)	25 (100%)	38 (100%)

Abbreviations: DLBCL, diffuse large B‐cell lymphoma; EBV, Epstein–Barr virus; NOS, not otherwise specified; PTLD, post‐transplant lymphoproliferative disorder.

^a^ Median (Q1–Q3 =).

^b^ Smooth muscle tumor is not PTLD but was included for this analysis.

## 4 Discussion

Our study is unique with respect to the large number of recipients monitored with standardized frequent EBV VL tests in the first post‐transplant year using protocols standardized across organ types and adult and pediatric high‐risk EBV‐mismatched recipients. This allowed more precise understanding of the incidence and timing of EBV DNAemia in this population. Studies that used plasma or did not specify the sample type used for VL monitoring were generally excluded as comparators. In our study, despite antiviral prophylaxis, the incidence of WB EBV DNAemia in the first post‐transplant year was high (49%) in EBV‐mismatched recipients, reaching cumulative incidences of 63% and 75% in kidney and liver recipients, respectively, when infant donors were excluded. This compares to a smaller study in adult D^+^/R^−^ recipients of mixed allograft type, documenting an incidence of 67.2% in the first year [15].

We found that DA‐EBV risk was associated with organ type. We observed the highest cumulative incidence of EBV DNAemia in liver recipients, confirming the high incidence of 82% within 2 years and 85% within 1 year reported in D^+^R^−^ pediatric liver populations [16, 17]. EBV DNAemia risk was lower in kidney compared to liver recipients in multivariable analyses, consistent with reports of mostly lower EBV DNAemia incidence in adult and pediatric kidney cohorts as follows: 63% by 1 year in D^+^R^−^ pediatric recipients reported by Höcker et al., 43%–58% in seronegative adult (Ville 2018) and 30% in seronegative pediatric recipients by 1 year (Yamada 2018), and 48.5% in seronegative pediatric recipients during longer follow‐up (Columbini 2017) [18, 19, 20, 21]. Ladfors et al. reported a higher incidence of 81% by 1 year in a seronegative pediatric kidney population [22]. The latter four studies included minor populations of low‐risk D^−^R^−^ patients (∼10% of cohorts) [19, 20, 21, 22]. In our study, DA‐EBV incidence was lower in heart compared with liver or kidney recipients; however, this decreased risk in heart recipients was not confirmed in multivariable analysis (donors and recipients <12 months excluded). A lower incidence of EBV DNAemia was also observed in pediatric heart versus liver recipients in the first year post‐transplant in a multicenter study with mixed allograft types and EBV D/R status; VL specimen type was unspecified [23]. We observed an interesting pattern in our common donors. When seropositive donors inconsistently transmitted to multiple recipients, the non‐infected recipient was the heart recipient, a donor transmission pattern also observed for CMV [24]. We were surprised by the lower risk of DA‐EBV in lung compared to kidney recipients. We believe this likely reflects lower compliance with VL monitoring in our lung population; this lower risk was lost in multivariable modeling when restricted to recipients with ≥50% monitoring compliance. Although EBV DNAemia is often prolonged after primary infection in SOT recipients, very infrequent monitoring may underestimate EBV infection. In a study of adult kidney recipients, the incidence of EBV infection increased by 29% when seroconversion was added to EBV DNAemia in the definition [19]. While reasons for organ‐specific differences in DA‐EBV are unknown, the relative load of latently infected B cells delivered with the graft may be an important factor. For EBV transmission to occur, there must be lytic reactivation of EBV from latently infected B cells in the donor organ leading to infection of recipient B cells. Reactivation event probability likely correlates with latently infected B‐cell load in the graft.

A striking observation in our study, not previously documented, was the very low risk of DA‐EBV in recipients less than 6 months old, regardless of donor age and the presence or absence of maternal EBV antibody, and in recipients less than 12 months old receiving organs from seropositive donors less than 12 months old. Data suggest that infants may be protected from natural EBV infection during the first 6 months of life [25, 26, 27]. The pathogenesis of this protection is uncertain. Although not directly related to levels of neutralizing antibody, in African infant cohorts, 100% of whom have maternal EBV antibody, the duration of protection appears dependent on quantitative maternal antibody levels and their decay kinetics [25, 28]. It is uncertain whether this protection extends to organ DA‐EBV infection, which is iatrogenic and bypasses normal mucosal protection barriers, and to infants less than 6 months old without maternal antibody. We documented only one case of DA‐EBV among 11 recipients less than 6 months old, three of whom had no pre‐transplant EBV antibody, who received organs from likely truly EBV‐infected donors over 12 months old. Risk appears to change after 6 months of age. Among 50 recipients 6–12 months of age with donors over 12 months of age, a similar incidence of DA‐EBV transmission was observed regardless of whether they were EBV seropositive or seronegative pre‐transplant (53.8% and 58.3%). This suggests that factors other than maternal antibody may be at play in providing protection from EBV infection in young infants, such as adequate maturation of EBV receptors or immature immune responses that decrease the probability of lytic EBV reactivation from latently infected graft B cells [29]. In contrast to African infants who become universally EBV‐infected between 6 and 24 months of age, a Swedish study found only 13% and 19% of children had seroconverted to EBV by age 1 and 2 years, respectively [28, 30]. Although influenced by ethnicity, it is likely that most seropositive donors less than 12 months old are not EBV‐infected, only falsely positive because of passive maternal antibody [31]. Assigning the “worst case scenario” to recipients less than 6 months and donors less than 12 months old may significantly overestimate DA‐EBV risk. Viral detection studies, including EBV DNA saliva testing, should be explored to clarify true EBV infection status of recipients and donors less than 12 months to inform more targeted EBV monitoring algorithms.

Interestingly, in recipients over 12 months old, we observed a decreasing risk of DA‐EBV with increasing recipient age. Potential explanations for this include higher loads of infected B cells relative to bodyweight with use of adult donors for young children, possible earlier destruction of infected B cells in older children and adults with more mature immune systems, and misclassification of early CA‐EBV infection as DA‐EBV, as CA‐EBV is likely less common in older adults.

To increase cost‐effectiveness and decrease the burden of EBV VL monitoring in EBV‐mismatched SOT recipients, the timing and frequency of monitoring should focus on the time of highest EBV DNAemia incidence. Our study suggests that delaying initiation of VL monitoring until 3–4 weeks post‐transplant is reasonable, after ruling out acute infection at transplant. Evidence suggests valganciclovir prophylaxis may delay and even prevent EBV infection post‐transplant, although this is not consistently documented [19, 20, 32, 33]. We cannot comment on the impact of anti‐viral prophylaxis in our study except to say that EBV DNAemia was frequently detected while recipients were receiving their antiviral prophylaxis of at least 14‐week duration; this has been observed by other researchers [19, 20, 34]. Our median time to DA‐EBV of 83 days compares to other estimates in the first post‐transplant year of 129 days in mixed allograft type D^+^R^−^ adults and 43 days in predominantly D^+^R^−^ seronegative pediatric kidney recipients [15, 22]. Longer median times to EBV DNAemia of 7.2 and 8.4 months have been described in seronegative pediatric kidney recipients, which included some D^−^R^−^ recipients with prolonged follow‐up, likely reflecting inclusion of later CA‐EBV infection [20, 21].

Our definition of DA‐EBV as transmission in the first year was arbitrary. We found that most EBV transmission occurs within 6 months. We observed a lower incidence of first EBV DNAemia from 6 to 12 months post‐transplant, which may be a mix of DA‐EBV and CA‐EBV. More precise ascertainment of the risk and patterns of CA‐EBV would require cohort studies using EBV DNAemia and serology in D^−^R^−^ cohorts.

In the most recent era, a decrease in PTLD incidence in the first post‐transplant year, possibly attributable to the implementation of pre‐emptive EBV VL‐based PTLD prevention strategies and changes in immunosuppression, has been described [5, 35]. Despite EBV VL surveillance, 32% of our PTLD cases occurred in the first post‐transplant year, similar to the 33% described in a national pediatric PTLD registry [36]. Our study suggests that more frequent EBV VL monitoring of EBV D^+^/R^−^ recipients in post‐transplant months 2–5, when the incidence of first DA‐EBV detection is highest, would maximize opportunities to intervene early in EBV DNAemia to prevent PTLD, consistent with recommendations in recent guidelines [11]. A possible exception is recipients less than 6 months and recipients 6–12 months with donors less than 12 months old who appear to be at lower risk of DA‐EBV. In our study, while nine of 40 recipients who were infants at low risk of DA‐EBV at transplant developed PTLD, only two of nine had detectable EBV VL within the first year post‐transplant, and the median time to PTLD in this group was quite long at 37 months. Other investigators have found a lower early PTLD incidence and longer time from transplant to PTLD in infants, predominantly heart recipients, compared with older children [37, 38, 39]. This highlights the importance of later CA rather than only DA‐EBV infection in this group. Focusing VL surveillance in infants at “low risk” for DA‐EBV at detecting CA‐EBV may be a preferred approach. This would be similar to strategies suggested in the IPTA consensus guidelines for EBV VL monitoring for EBV D^−^R^−^ cohorts, with less intensive monitoring in the first 6 months when blood‐taking burden is already high, but continued monitoring beyond 1 year post‐transplant [11]. Our observations suggest this may be a safe and practical option in this infant population.

In our study, a significant proportion (14.8%) of recipients developed PTLD, with the rate particularly high in adult lung recipients at 36%. This is similar to the 17.6% found in D^+^R^−^ adults, but higher than the 6.8% reported in D^+^R^−^ children [15, 23]. Proportions of allograft type and follow‐up duration may account for the differences observed. Most cases of PTLD with EBER testing performed were EBER‐positive (90.6%). WB EBV VL detection has a low positive predictive value, but is generally thought to have a high negative predictive value for diagnosing EBER‐positive PTLD [11, 40, 41]. However, we found five recipients with EBER‐positive PTLD who never had a detectable EBV VL; all had a negative EBV VL within 15 days of diagnosis. EBV‐positive PTLD in the absence of detectable EBV DNA in the peripheral blood has been described when PTLD is limited to immune‐privileged sites including the central nervous system (CNS) and testes, in mucocutaneous ulcer and in thoracic recipients with disease limited to the lung, but this was not the case for three of the five recipients in our study [41, 42, 43, 44, 45]. This warrants further study, but our findings certainly suggest that a negative WB EBV PCR should not be used to rule out EBER‐positive PTLD.

Our study had limitations. It was a single‐institution retrospective study. There may have been misclassification of EBV serostatus in infant donors and recipients less than 12 months old due to passive maternal antibody. Some donors may have been falsely seropositive due to blood products; transfusion data were not available. As EBV VL monitoring frequency decreased after 5 months post‐transplant, we may have missed some DA‐EBV. This seems unlikely as VL monitoring still occurred monthly and EBV DNAemia commonly persists for many months in this population. Incidence of DA‐EBV may have been underestimated in lung and heart recipients with lower monitoring compliance. Our WB testing data cannot be extrapolated to plasma testing. Although centers following patients remotely were asked to report PTLD cases back to our center and compliance is perceived to be good, compliance was not audited and cases may have been unreported.

## Conflicts of Interest

The authors declare no conflicts of interest.

## Supporting information



Supporting Information

## Data Availability

Data are available on request from the authors.
