# Supplementary material for: Incidence and Timing of Epstein–Barr Virus Whole Blood DNAemia in Epstein–Barr Virus‐Mismatched Adult and Pediatric Solid Organ Transplant Recipients
Source: Transpl Infect Dis. 2025 Apr 29;27(4):e70042. doi: 10.1111/tid.70042 (PMC12416345; doi:10.1111/tid.70042)

Supplementary Table S1. **Immunosuppression Administered at our Center by Organ Group**

| Organ | Induction | Maintenance |
| --- | --- | --- |
| Adult and Pediatric Kidney | Thymoglobulin (2000-2018) for highly sensitized. Low immunologic risk pts given IL2-receptor antagonists Daclizumab (2000-2004) or Basiliximab (2005-2018). | Triple therapy throughout study period, using Tacrolimus, MMF and steroids, tapered to steady state levels by the end of the first post-transplant year. Sirolimus used only in CNI toxicity. |
| Adult and Pediatric Liver | Daclizumab (2002-2009)  Basiliximab (2009-2018) | Tacrolimus and MMF with steroid withdrawal (2002-2018); pts with HCC (25%) or renal dysfunction converted within 4-12 weeks to sirolimus and MMF (HCC) or sirolimus and low dose tacrolimus, with the goal of sirolimus monotherapy by one year if rejection-free. |
| Adult Heart and/or Lung | RCT ATGAM vs. Daclizumab (2001-2005 heart, 2001-2003 lung)  Heart recipients received ATGAM (2005-2011) or Thymoglobulin (2011- 2018)  Lung recipients received Daclizumab (2003-2011)/ Basiliximab (2011-2018) or ATGAM (2003-2018) at the physician’s discretion | Triple therapy throughout study period, using Tacrolimus, MMF and steroids. Sirolimus used only in CNI toxicity. |
| Pediatric Heart | (2000-2010) ATG  (2011-2018) ATG for high risk, otherwise Basiliximab for most  (2010-2016) unsensitized ABO compatible infant transplants received no induction, since 2016 received Basiliximab | Tacrolimus with MMF and steroids initially. Steroids weaned at 3-6 months post-transplant. |
| Adult and Pediatric Intestinal /  multi-visceral | Thymoglobulin (2003-2018) | Triple therapy throughout the study period with low-dose tacrolimus, sirolimus and steroids tapered to steady state levels over one year |

CNI = calcineurin inhibitor, HCC = hepatocellular carcinoma, MMF = mycophenolate mofetil, pts = patients.

**Supplementary Table S2.** **Demographics of EBV mismatched (EBV D+/R-) recipients by organ group**

|  | **N of Kidney recipients (%)** | **N of Liver recipients (%)** | **N of Heart recipients (%)** | **N of Lung recipients (%)** | **N of All recipients (%)** |
| --- | --- | --- | --- | --- | --- |
| **Recipient Age** |  |  |  |  |  |
| <6M | 0 (0.0%) | 5 (5.4%) | 24 (32.0%) | 0 (0.0%) | 29 (11.3%) |
| 6M-12M | 0 (0.0%) | 45 (48.4%) | 16 (21.3%) | 0 (0.0%) | 61 (23.7%) |
| 12M-5y | 8 (12.5%) | 17 (18.3%) | 10 (13.3%) | 0 (0.0%) | 35 (13.6%) |
| 5y-17y | 13 (20.3%) | 11 (11.8%) | 13 (17.3%) | 0 (0.0%) | 37 (14.4%) |
| ≥17y | 43 (67.2%) | 15 (16.1%) | 12 (16.0%) | 25 (100.0%) | 95 (37.0%) |
| **Donor Age** |  | | | | |
| <12M | 0 (0.0%) | 8 (8.6%) | 23 (30.7%) | 0 (0.0%) | 31 (12.1%) |
| ≥12M | 64 (100.0%) | 85 (91.4%) | 52 (69.3%) | 25 (100.0%) | 226 (87.9%) |
| **Recipient Sex** |  | | | | |
| Female | 22 (34.4%) | 42 (45.2%) | 28 (37.3%) | 12 (48.0%) | 104 (40.5%) |
| Male | 42 (65.6%) | 51 (54.8%) | 47 (62.7%) | 13 (52.0%) | 153 (59.5%) |
| **Total Recipients** | 64 (100.0%) | 93 (100.0%) | 75 (100.0%) | 25 (100.0%) | 257 (100.0%) |
| **Median % (Q1-Q3) Compliant with follow-up** | 100% (87.5-100) | 100% (75.0-100) | 66.7% (29.2-62.8) | 29.2% (8.3-87.5) | 91.6% (58.3-100%) |

**Supplementary Figure S1. Time to first detectable whole blood EBV DNAemia during the first post-transplant year in EBV D+/R- SOT recipients (N=126)**

**
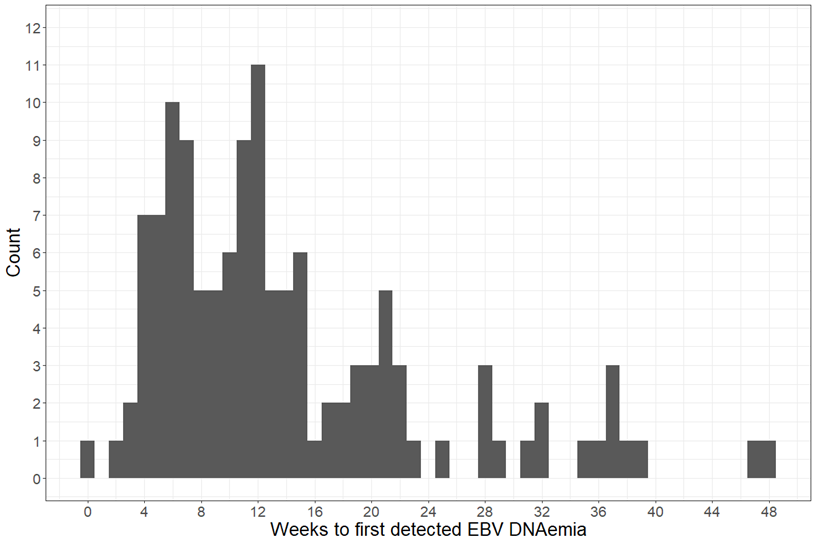
**

**Supplementary Figure S2a.** **Timeline of EBV Viral load testing and PTLD Diagnosis: 9 cases with no detectable whole blood EBV DNAemia prior to/at the time of PTLD Diagnosis.**

Case details include: organ transplanted, PTLD EBV- encoded small RNAs (EBER) status (Positive (+), Negative (-), Unknown (?)), and Age at transplant (in years). *Age 10 days, ** Age 0 days.


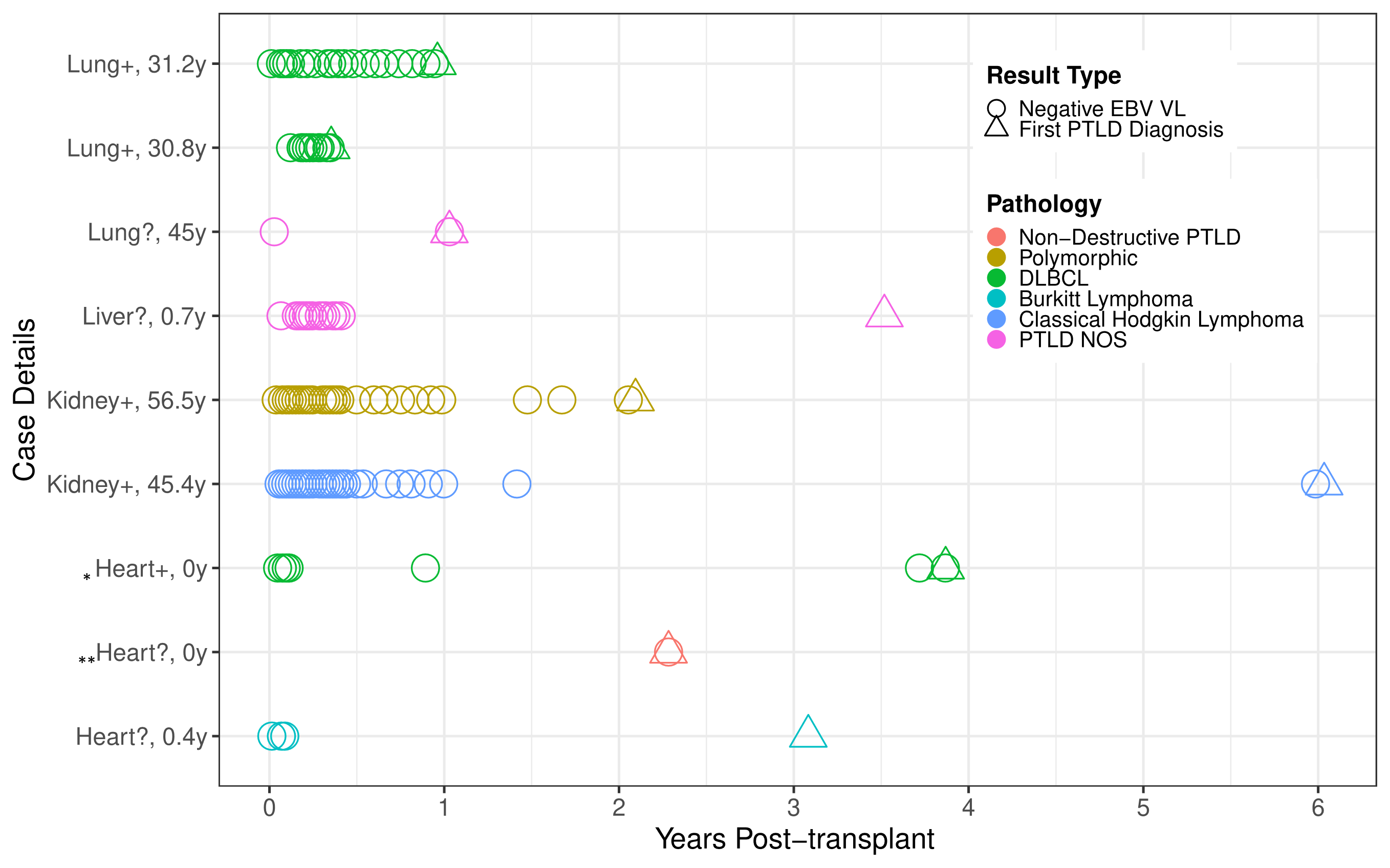


**Supplementary Figure S2b.** **Timeline of EBV Viral load testing and PTLD Diagnosis: 29 cases with detectable Whole Blood EBV DNAemia prior to/at time of PTLD diagnosis**

Case details include: organ transplanted, PTLD EBV- encoded small RNAs (EBER) status (Positive (+), Negative (-), Unknown (?)), Age at transplant (in years) and time from first detectable EBV VL to PTLD diagnosis in years. Time from first EBV detection to PTLD diagnosis includes first EBV detection up to 4 weeks post-diagnosis of PTLD – these values are adjusted and reported as 0 in the figure for cases marked with *

‡Smooth Muscle Tumor is not true PTLD but included in our analysis


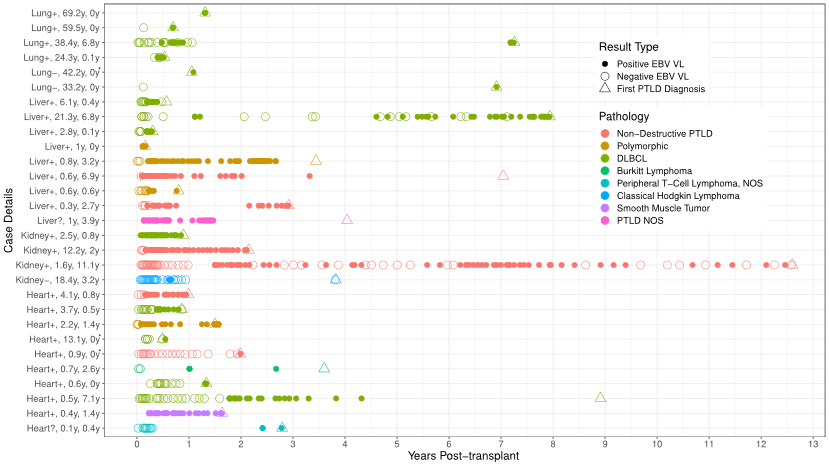

Supplement: Supplementary file 1 — Supporting Information [file TID-27-e70042-s001.docx]
